# Supplementary figures and images for: Phage production is blocked in the adherent-invasive Escherichia coli LF82 upon macrophage infection
Source: PLoS Pathog. 2023 Feb 2;19(2):e1011127. doi: 10.1371/journal.ppat.1011127 (PMC9928086; doi:10.1371/journal.ppat.1011127)

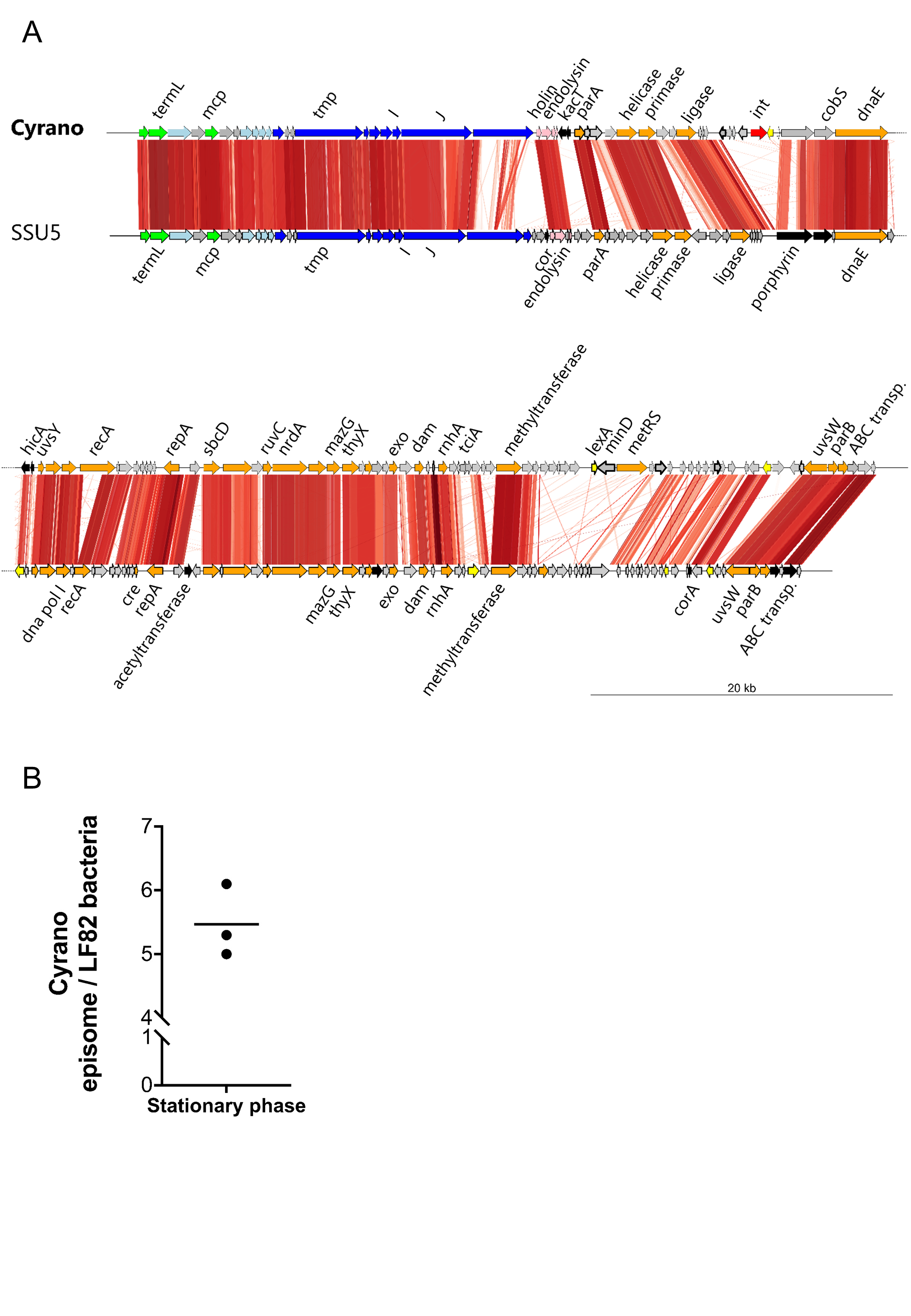

Supplement: S1 Fig — A. Whole genome comparison of the LF82 phage-plasmid Cyrano and SSU5. A tBLASTx comparison was performed and visualized with the R package Genoplot. The heat map and gene color indications used here are the same as those used for Fig 1. B. Determination by qPCR of the Cyrano copy number per E. coli LF82 bacteria. Each dot corresponds to one biological replicate. The mean of these values (5.5) is represented by a vertical line. (TIF) [file ppat.1011127.s001.TIF]

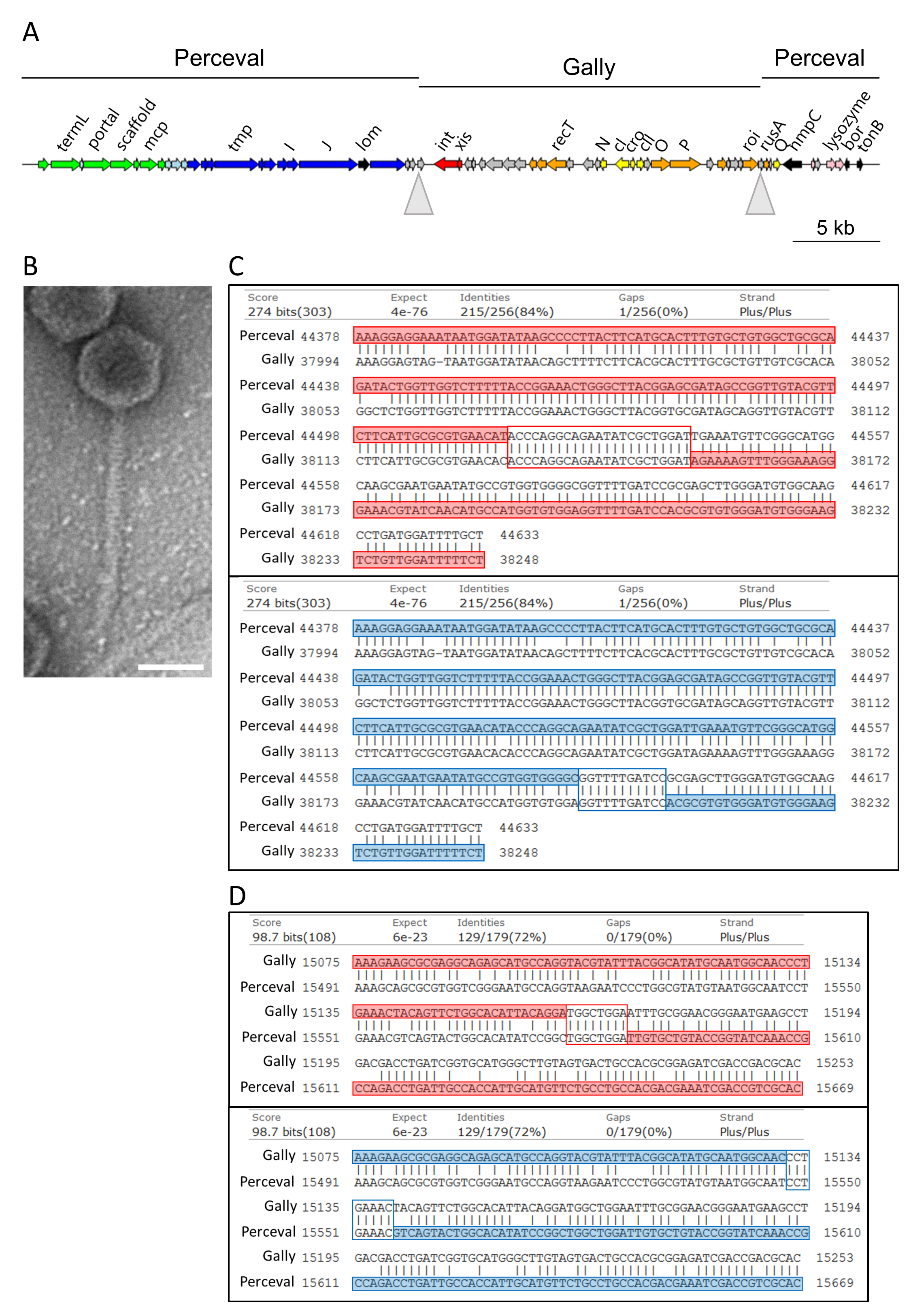

Supplement: S2 Fig — A. Genetic map of the Galper hybrids. Grey triangles indicate the two recombination endpoints between Gally and Perceval. B. Transmission electron microscopy photograph of the purified Galper1. Scale bar is 50 μm long. C and D. Sequence analysis of the first (C) and the second (D) recombination endpoints in Galper1 (upper panels, red) and Galper2 (bottom panels, blue), which occur respectively in a 256 and a 179 bp region of partial homology between Perceval and Gally ((C) 84% identity, (D) 72% identity). (TIF) [file ppat.1011127.s002.TIF]

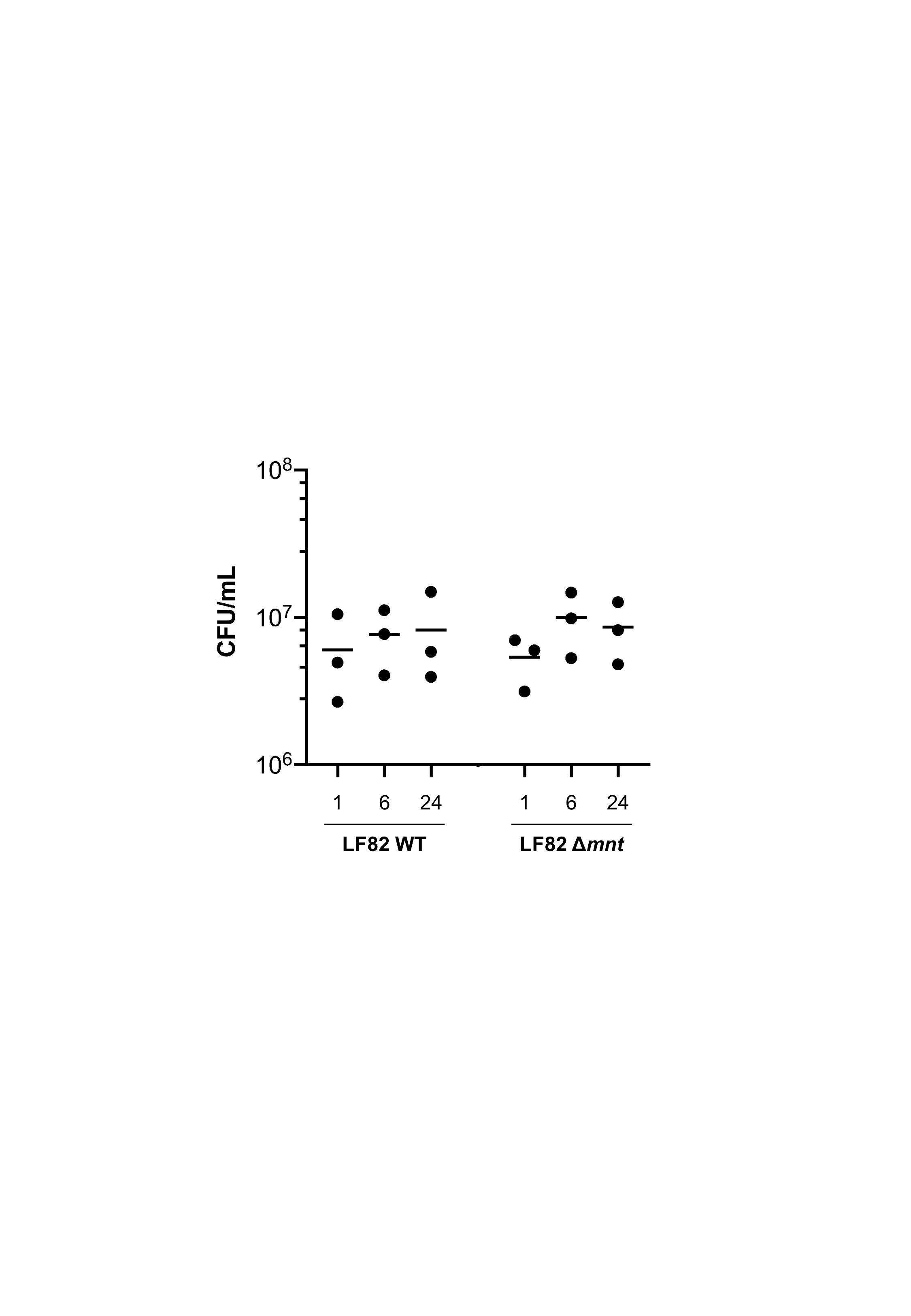

Supplement: S3 Fig — Each dot corresponds to a biological replicate, from an independent macrophage infection. Horizontal black lines represent mean values. (TIF) [file ppat.1011127.s003.TIF]

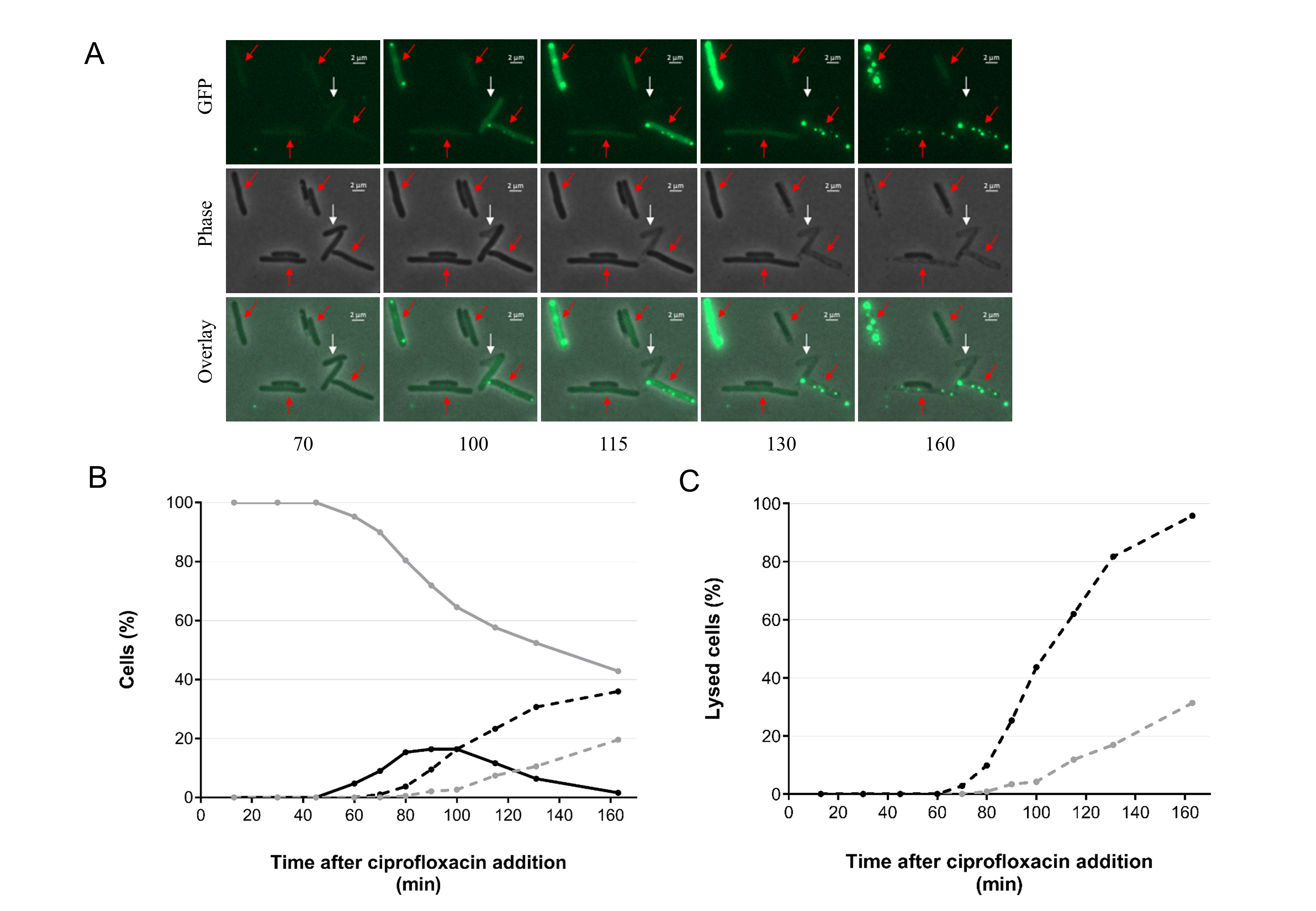

Supplement: S4 Fig — A. Fluorescence (GFP) and phase channel images obtained at different time points after deposition are shown, along with an overlay of these images. Red arrows: cells becoming fluorescent and lysing during incubation. White arrow: lysed cell without MCP-GFP induction. B. Quantification of the four categories of cells monitored: intact or lysed non-fluorescent cells (solid or broken gray lines, respectively), and intact or lysed fluorescent cells (solid or broken black lines, respectively). C. Cell lysis over time as a function of the prior induction (black dashed line) or not (gray dashed line) of MCP-GFP fusion protein expressed from Gally phage. (TIF) [file ppat.1011127.s004.TIF]

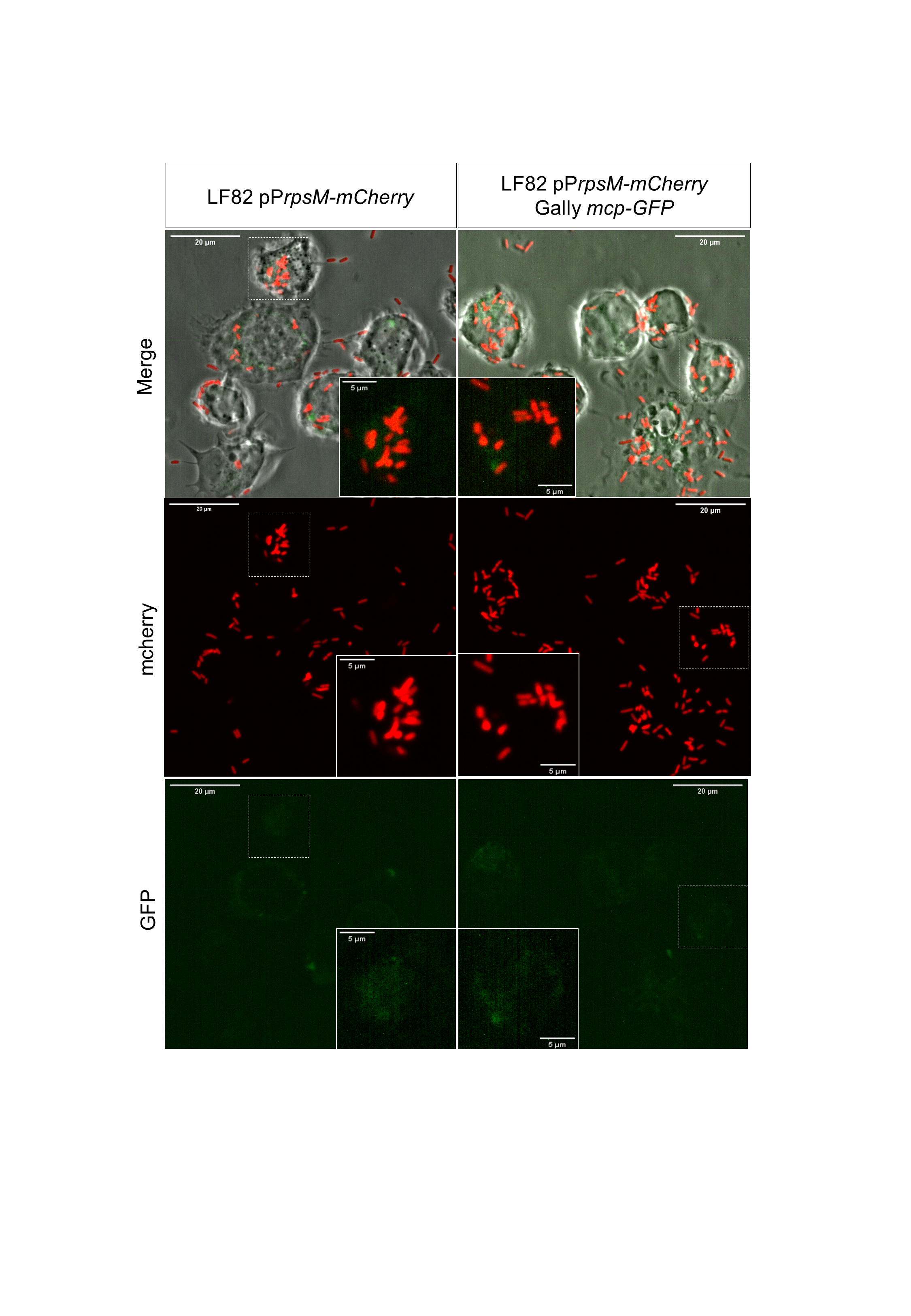

Supplement: S5 Fig — (TIF) [file ppat.1011127.s005.TIF]

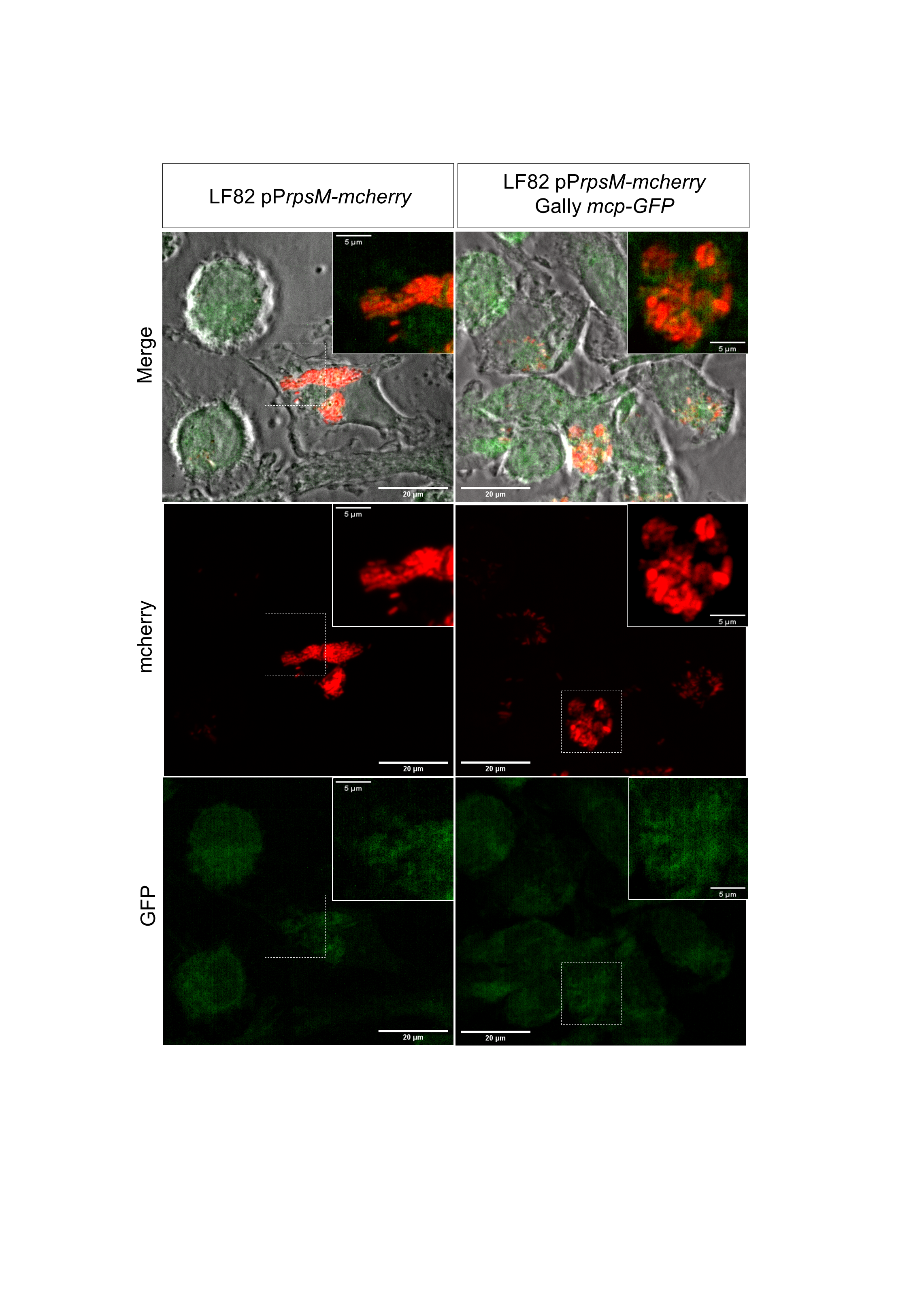

Supplement: S6 Fig — (TIF) [file ppat.1011127.s006.TIF]

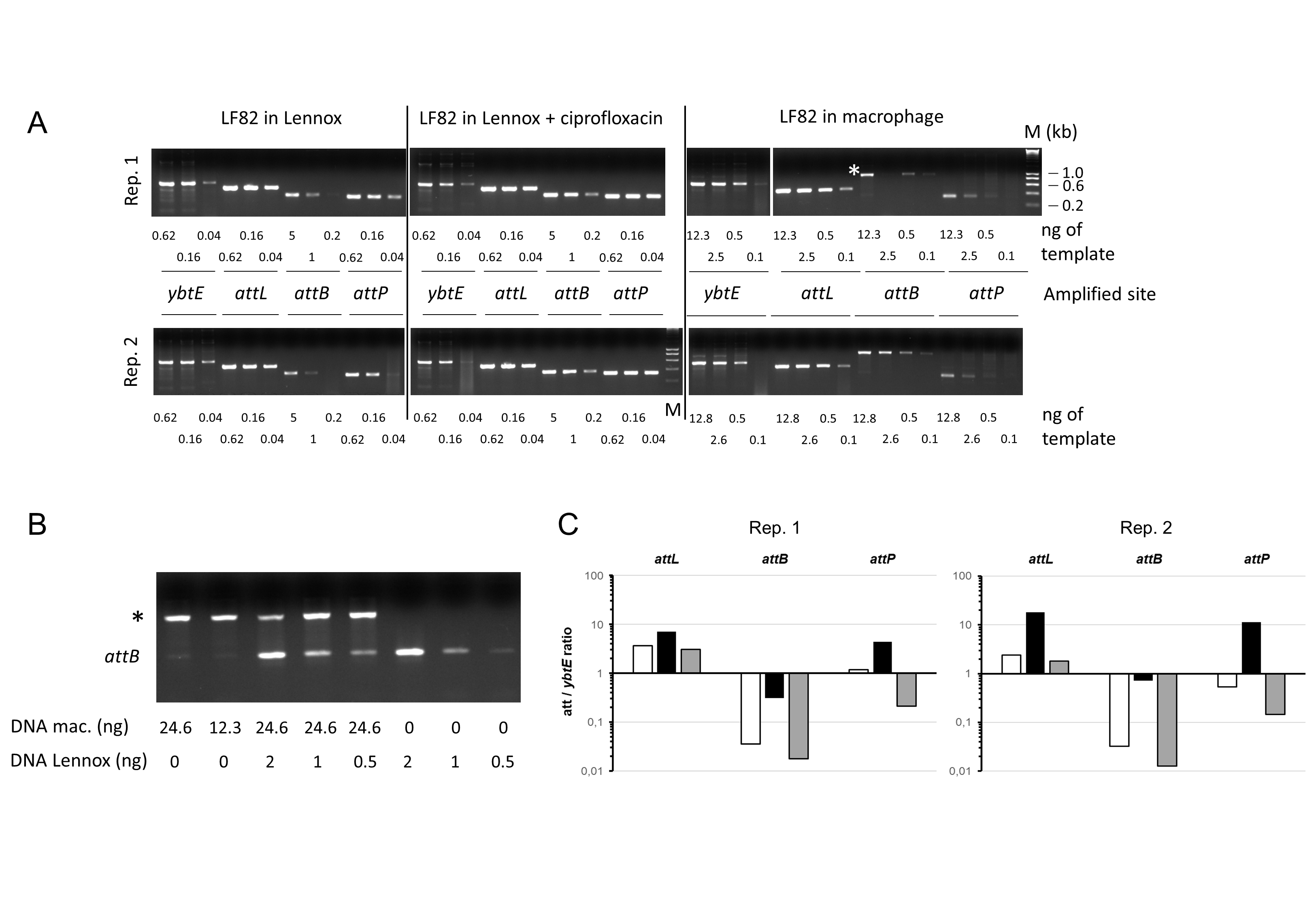

Supplement: S7 Fig — A. PCR amplification product analyzed by gel electrophoresis in the presence of ethidium bromide, obtained with the following oligonucleotide pairs: OPM80/OPM82 (ybtE), OPM75/Maj281 (attL), JC206/JC207 (attB) and Maj280/Maj281 (attP). The DNA templates used (quantities indicated below the gels) were purified from LF82 bacteria grown either in Lennox medium, Lennox with ciprofloxacin (at the MIC) for ~1 hour, or within macrophages for 6 hours, as indicated. Two replicates were analyzed for each condition. M: molecular weight marker. The asterisk denotes a contaminant amplified product obtained with the DNA template from LF82 bacteria grown in macrophages. B. Evaluation of the impact of contaminant amplification (*) on the amplification of the Gally attB site. The excision site was amplified from mixes of the indicated amounts of purified LF82 DNA template extracted from bacteria grown in macrophages (DNA mac.) or Lennox (DNA Lennox) and analyzed by agarose gel electrophoresis. Amplification of the contaminant product does not repress amplification of the attB site. C. Ratio of the different att sites over the bacterial ybtE gene, used as a reference, in unstressed (white bars) or stressed (ciprofloxacin, black bars) in vitro growth conditions and in macrophages (6 hours P.I., grey bars). Bands on the gel were quantified using Image Lab software for both sets of replicates. Except for some attB ratios, ratios were computed by dividing att site amounts generated from a defined input of template DNA, by ybtE amounts generated from the same DNA input. For attB under Lennox (+/- cip) growth conditions, the attB/ybtE ratio was calculated by dividing the amount of attB PCR products obtained from 5, 1, and 0.2 ng of template DNA by the amount of ybtE amplified product from 0.62, 0.16, and 0.04 ng, respectively. The ratios obtained were then multiplied by the difference in the amount of template DNA used for PCR for attB and ybtE. (TIF) [file ppat.1011127.s007.TIF]
