# Supplementary material for: Phage production is blocked in the adherent-invasive Escherichia coli LF82 upon macrophage infection
Source: PLoS Pathog. 2023 Feb 2;19(2):e1011127. doi: 10.1371/journal.ppat.1011127 (PMC9928086; doi:10.1371/journal.ppat.1011127)
Supplement: S3 Table — (+)*: virions quantification contaminated by Gally-mediated lateral transduction. ND: not determined. (PPTX) [file ppat.1011127.s010.pptx]

## Slide 1
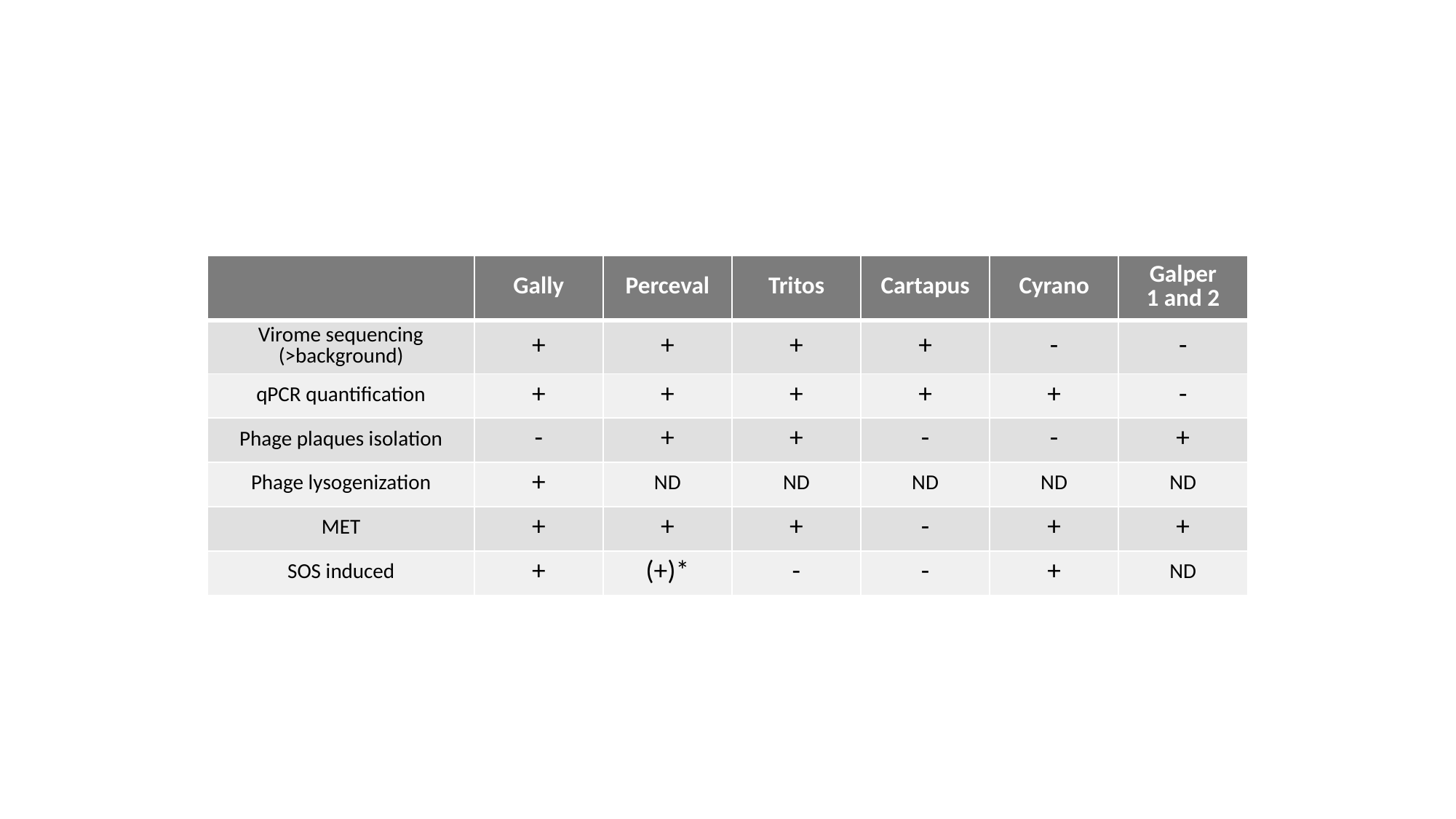

| | Gally | Perceval | Tritos | Cartapus | Cyrano | Galper 1 and 2 |
| --- | --- | --- | --- | --- | --- | --- |
| Virome sequencing (>background) | + | + | + | + | - | - |
| qPCR quantification | + | + | + | + | + | - |
| Phage plaques isolation | - | + | + | - | - | + |
| Phage lysogenization | + | ND | ND | ND | ND | ND |
| MET | + | + | + | - | + | + |
| SOS induced | + | (+)\* | - | - | + | ND |
