# Supplementary material for: Phage production is blocked in the adherent-invasive Escherichia coli LF82 upon macrophage infection
Source: PLoS Pathog. 2023 Feb 2;19(2):e1011127. doi: 10.1371/journal.ppat.1011127 (PMC9928086; doi:10.1371/journal.ppat.1011127)
Supplement: Attachment — Submitted filename: response to reviewers.docx [file ppat.1011127.s012.docx]

## 25th November 2022

Institut MICALIS, INRAE

AgroParisTech, Université Paris-Saclay

F-78350 Jouy-en-Josas, France

Tel: (33) 1 34 65 20 81

Fax: (33) 1 34 65 25 21

E-mail: francois.lecointe@inrae.fr

To the editors of PLOS Pathogens

Object: Revised version of manuscript PPATHOGENS-D-22-00340

Please find the revised version of our manuscript in which we have carefully considered all the comments made by the three reviewers, including the addition of several new experiments characterizing, among others, the effect of Gally induction on LF82 viability, the impact of the macrophage on the replication and excision steps of the Gally genome and the ability of Gally to lysogenize a strain lacking the prophage.

Below is a list of our point by point responses (in blue) to all the reviewers’ comments. We included most of the suggested additional assays, figures, analyses and corrections. For clarity, the changes are indicated in red characters in the revised manuscript.

We would like to thank the reviewers for constructive criticism of our manuscript and for their helpful suggestions and questions that have greatly improved the quality of the article.

With my best regards,

Francois Lecointe

We apologize for the very long time it took to secure reviews for this work. Unfortunately three independent scientists have reviewed the work and all have significant concerns. The associate editor has also reviewed the manuscript and concurs with some but not all of the reviewer's criticisms. We cannot accept the manuscript as is but would be willing to consider a substantially revised work that addresses the reviewers primary concerns.

The associate editor does not feel that one comment from reviewer 1 needs to be addressed directly and that there may be some confusion in that reviewer's logic. The fact that a Gally knockout in bacteria is equivalent to a repressed prophage makes sense and is not contradictory. The associate editor does agree that a shortcoming is a lack of a clearly proposed mechanism for how prophages are repressed in the macrophage. Reviewer 3 has several concerns about methodologies and controls that need to be addressed.

Reviewer's Responses to Questions

**Part I – Summary**

Please use this section to discuss strengths/weaknesses of study, novelty/significance, general execution and scholarship.

**Reviewer #1:**

In this manuscript, the authors thoroughly characterized 5 prophages in the adherent-invasive E. coli LF82. The numbers of viral genomes in the culture supernatant were determined, and the morphology was also determined for four phages (except for Cartapus). For Gally, the most highly induced prophage, evidence for lateral transduction was observed. The deletion of Gally did not affect bacterial survival during the infection of macrophages. Contrary to the expectation, the induction of prophages, including Gally, was reduced in the macrophages. In the case of Gally, some parts of the prophage genome appeared repressed during the infection of macrophages. Based on these results, the authors concluded that the E. coli strain actively controls the induction of the most active prophage Gally, which might enhance the bacterial survival in macrophages.

This manuscript is well-written with high clarity. In general, science is very solid, and I do not have any serious concerns about the methodology. However, it is difficult to accept the authors’ conclusion. The authors demonstrated that Gally plays no significant role in the bacterial survival in macrophages. Then, how could reducing induction of the prophage help bacteria survive better in macrophages? Since the induction of other prophages is also reduced, it is possible that the intracellular environment of macrophages has a low inducting activity for the prophages, rather than the LF82 strain actively reducing the prophage induction? Finally, no reasonable mechanistic model was provided for the selective repression of Gally genes.

We thank the reviewer for this comment. We would like to emphasize that, based on published data on lysogens, and previous reports on SOS induction in macrophages, we were expecting a drastic prophage induction in macrophage, and hence, a much lower survival of LF82 compared to its Gally-deleted isogenic strain. We show there is no difference in survival between these two strains, so this leads us to investigate further how is the prophage cycle controlled in this environment. This new version of the manuscript enters into further details with respect to the mechanism controlling Gally induction.

Concerning the mechanism of control and as described below, we assessed the importance of the gene encoding a putative repressor homolog to the P22 Mnt, which is overexpressed within macrophages. We found no effect on the viability of a Δ*mnt* strain compared to the wild type. Finally, transcriptomic data and quantification of replication *in situ*, excision, and replication post-circularization of the Gally genome in macrophages, added in the revised version (see below), suggest a control mechanism of the Gally lytic cycle, repressing the prophage excision specifically in macrophage. However, the molecular basis of this mechanism remains to be characterized, which will be the subject of a future study.

**Reviewer #2:**

In this study, the authors performed detailed analyses of 5 active prophages of E. coli strain LF82, the prototype AIEC strain. They isolated two phages, visualized the 4 phages, and identified moron genes. As for the most active one (named Gally), they provide evidence of lateral transduction and formation of hybrid phages and show that Gally (and other phages) do not produce much lower numbers of virion in macrophages. I appreciate the authors' efforts, especially those to isolate and visualize the five phages and to provide clear evidence of lateral transduction of Gally. But, a weak point of this study is that, regarding the suppression of prophage in macrophages, it is not clear what happened in macrophages, although I understand this is a difficult challenge because the prophage induction that the authors tried to analyze occurred only in a very small portion of bacterial cells.

We thank the reviewer for this comment. The particular transcriptional profile of Gally genes within macrophages suggests a regulatory mechanism that blocks the lytic cycle of the phage after its induction. Among the putative transcriptional regulators encoded by Gally, we evaluated, during the revision process, the impact of deletion of the gene coding for the Mnt protein, displaying homology to the P22 Mnt repressor of the lytic cycle, on LF82 survival in macrophages. We demonstrate that Gally virion production does not appear to be Mnt-dependent as the survival of the wild-type and Δ*mnt* strains in macrophages were similar (see below). However, thanks to the reviewer request, we performed semi-quantification of the replication *in situ*, excision, and replication post-circularization of the Gally genome in macrophages, highlighting what happened in macrophages concerning Gally, and focusing the step that is controlled in macrophages, even if the molecular mechanism controlling Gally induction remains to be characterized.

**Reviewer #3:**

The authors report on experiments aimed at characterizing 5 prophages in the genome of an AIEC strain. They use HT sequencing, transcriptomics, microscopy and a fluorescent reporter of lytic gene expression to examine liberation, phage lifestyle and morphology in response to antibiotics and macrophage infection. Unfortunately, the narrow range of methodologies applied, the lack of controls and experimental description make it impossible to validate the very compelling proposal that titles the paper, namely that prophage liberation is tamed upon macrophage infection.

As described below, we have tried to address all the constructive criticism made by Reviewer 3. We really appreciate the thorough revision made by the reviewer which has greatly, in addition to the other remarks made by reviewers 1 and 2, improved the quality of the article.

**Part II – Major Issues: Key Experiments Required for Acceptance**

Please use this section to detail the key new experiments or modifications of existing experiments that should be absolutely required to validate study conclusions. Generally, there should be no more than 3 such required experiments or major modifications for a "Major Revision" recommendation. If more than 3 experiments are necessary to validate the study conclusions, then you are encouraged to recommend "Reject".

**Reviewer #1:**

1. To validate the conclusion, the authors need to show the relevance of Gally induction to the survival of the bacterium.

The reviewer is quite right to raise this important point. We focused on Gally because of its greater induction compared to other phages after genotoxic stress, suggesting that most of the cell lysis that happens after such treatment should be due to its induction. To validate this hypothesis, we added an experiment to measure the viability of the wild type strain after addition of ciprofloxacin at MIC in the culture medium and compared it to that of the ΔGally mutant. The result is presented in the revised Figure 6 panel A and shows a rapid and massive lysis of the wild type 50 min after addition of the drug, not observable in the Gally mutant strain. The lysis of cells after induction with ciprofloxacin is therefore mainly due to the Gally induction. A paragraph describing this new result, the new panel A in Figure 6 and the corresponding Material and Method section, have been added in the revised manuscript.

2. Also, the authors need to provide experimental evidence that bacteria, not the intracellular environment of macrophage, control the transcription of Gally.

The point raised by the reviewer is quite correct: we did not prove that the bacterium controls the induction of its prophages in the macrophage, but only that production of its two main genotoxic stress-inducible virions (Gally and Cyrano) is repressed upon macrophage infection. As the reviewer points out, the molecular mechanism of this repression was not identified in this study and could indeed be mediated by the intracellular environment of the macrophage (however known to be genotoxic and to induce SOS response in phagocytosed LF82 bacteria), the bacteria in this environment or by the prophages themselves. This is an important point but the control of Gally induction in this environment is clearly not trivial. Indeed, as we have shown in the first submitted version, the expression of several genes encoding key phage structural proteins is downregulated inside macrophages compared to LB *in vitro* culture. This suggests that the transcription unit encoding these proteins may be regulated by a repressor specifically expressed in this environment. We searched *in silico* for Gally-encoded repressors by identifying genes expressing putative DNA-binding proteins that are upregulated in macrophages compared to LB culture, and found a putative homolog of the P22 Mnt repressor which might be a good candidate. We tested the role of this putative repressor inside macrophages by deleting the Gally *mnt* gene and comparing the survival of the mutant with the wild type strain in this environment (New S3 Fig). The result indicates that the absence of *mnt* does not alter the survival of the bacteria in the macrophage for 24 hours after infection. The mechanism by which the Gally lytic cycle is repressed in macrophages remains to be identified. Not knowing the reason why Gally is repressed in the macrophage, we chose to change the title and running title of the article.

**Reviewer #2:**

The authors concluded that prophage induction is suppressed in macrophages. This is the major and most important finding of this study. But, as mentioned above, it is not clear what happened in phagosomes of macrophages. I have two major concerns related to it. Both are particularly important if the authors consider a mechanism/phenomenon similar to the active lysogeny observed for a Listeria prophage, phi10403S (P18, the last line~P19, L1-L4, L7-L11).

1. Virion production was investigated, but the authors did not examine the excision and replication of Gally genome, which are important hallmarks of prophage induction.

To examine excision, replication, and circularization of the Gally genome, we performed semi-quantitative PCR to estimate the amount of *attB*, *attL*, and *attP* sites and compared them to the bacterial *ybtE* gene, which was used as a reference (the regions surrounding these sites did not allow for the design of primers compatible with qPCR analysis). The DNA templates used were obtained from bacteria grown for 6 hours in macrophages or from cells grown *in vitro* in exponential growth in Lennox medium supplemented or not with ciprofloxacin (the *in vitro* conditions were used as controls). The results obtained (S7 Fig) indicate that *in situ* replication (*attL*), excision (*attB*), and replication post-circularization (*attP*) of Gally were higher in cells cultured with ciprofloxacin-supplemented medium compared to untreated controls, as expected. In macrophages, excision and post-circularization replication of the Gally genome were decreased compared to unstressed *in vitro* conditions, whereas *in situ* replication of the prophage was similar. These results were added in the revised manuscript and suggest that the lytic cycle of Gally is repressed differently in macrophages and in Lennox medium, with a blockage of the lytic cycle at the excision stage in macrophages. We are grateful to the reviewer for requesting these additional experiments that show that Gally does not perform active lysogeny in macrophages, which is consistent with the lack of effect of the phage deletion on bacterial viability in this environment. The discussion has been modified to take these new results into account.

2. Regarding the interpretation of RNA-seq data interpretation, it is difficult for me understand how the up or down-regulated gene clusters are related to the transcription units of Gally. I think the authors can show how the transcription units are organized in Gally in its lytic cycle.

In the revised manuscript, we have modified the Figure 7 to show the mapping of RNA-seq reads across the Gally genome, instead of the normalized expression per gene. This should facilitate the visualization of transcriptional units and non-coding RNAs, that may be involved in regulation of the Gally lytic cycle. Normalized expression per gene is now available in the new S4 Tab for all bacterial and phage genes under all conditions described in Prudent *et al*. 2021, as requested by the reviewer (see item 9, part III). RNAseq data described in Prudent *et al*. have been reprocessed to include Cyrano, extract coverage along the genome and perform statistical analysis of expression differences between MB6 and BLB.

Ideally, Cyrano should be analyzed in parallel as this phenomenon does not seem to be Gally-specific, although analyses of Cyrano is not so easy due to its low induction and low gene expression.

In the revised manuscript, we reassessed the number of Gally and Cyrano virions produced in macrophages, as we found that Gally virions were not stable in macrophage lysate (see response to reviewer 3, item 11, part II). Our new analyses did not allow to quantify Cyrano virions, due to a bacterial DNA contamination that was too high to identify Cyrano DNA included in capsids (Figure 8 panel A). We no longer discuss Cyrano in macrophages in the revised version, however normalized gene expression of Cyrano is available in the S3 and S4 Tab.

**Reviewer #3:** Additional comments:

1. What is the rationale for the use of a 5-fold increased expression above local genes as an identifier for morons? It seems that the authors should also determine whether these genes are part of other lysogenic gene clusters/operons and whether their expression is truly independent of other phage genes to identify them as morons.

We would like to mention that Raw transcriptome data have been reprocessed (see below) which led to slightly less morons identified. Using the newly normalized gene expression available in the S3 Tab, we show that the distribution of transcribed gene abundances in the prophage regions is bi-modal, with most genes being in the first peak (low abundance, repressed genes), and few genes in the second half of the distribution (highly transcribed, morons; see Fig below, Figures are also in S3 Tab). We indicate on this figure where the chosen cut-off stands (red bars). It corresponds always to at least 5 times the standard deviation of the first gene group abundance. Compared to the initial table of the morons, we have now decided to use the LB dataset only to define the moron genes, as we realized that DMEM, the medium used for macrophage growth, induces a few additional genes for some prophages. Clearly, morons have to be defined in a given condition and should depend on the condition analyzed. We decided to present only LB dependent morons in the revised manuscript.

Red lines : 5x median of local normalized read counts

2. In the first results section the authors state “Inspection of attachment sites of the four integrated prophages did not reveal any particular gene inactivation pattern”. Can they be more specific here? What were they looking for? What are the attachment sites?

We looked for a potential modification of the content of ORFs in the vicinity of the prophage attachment sites after their excision. Some prophages inactivate genes and their excision allows the reconstitution of an active ORF, which may alter the physiology of the bacterium (e.g., the disruption of the sporulation-related gene, *spsM*, by the prophage Spβ from *B. subtilis* (DOI: 10.1371/journal.pgen.1004636) or the *comK* gene disrupted by the Phi1403S phage from *Listeria monocytogenes*, which is at the origin of the active lysogeny mechanism (DOI: 10.1016/j.cell.2012.06.036)). Because our study suggests that LF82 has found a way to control its prophages in the genotoxic environment of macrophages rather than inactivating them, we asked whether LF82 retains active prophages to control bacterial genes that might be inactivated by prophage insertion.

To do this, we generated *in silico* a LF82 genome devoid of its prophages, thanks to the identification of prophage boundaries (Table 4). These boundaries were determined thanks to the virome sequencing and allowed us to deduce the putative *attB* sites of each prophage, on the LF82 chromosome. The *attB* site from Gally was also verified by sequencing of the corresponding region in the *E. coli* LF82 ΔGally mutant. This has been explained in the material and method section of the revised manuscript. The absence of reconstituted ORFs (>300bp) after prophage excision indicates that the chromosomal prophages of LF82 do not alter the bacterial gene content after their integration or excision.

For a sake of clarity, the paragraph lines 7-14 page 9 of the submitted manuscript has been modified and moved to lines 19-24 page 10 of the revised manuscript. During the revision of the manuscript we found errors in the coordinates of the prophage boundaries given in the submitted manuscript. These coordinates have been corrected in the revised version following the methodology detailed in the material and methods.

3. In Figure 2, in order to characterize phage release, the authors sequence DNase treated supernatants where they have destroyed capsids. The results for Gally are very convincing, however the results arguing that the other phages are spontaneously released are less so. A much narrower range of chromosomal background reads is shown for the other phages, and the phage signals appears to be much weaker. These things combined make it difficult to Perceval, Cartapus and Cyrano phages are present in the supernatant from cultures of E. coli LF82.

We have added a statistical analysis in the revised manuscript showing that all the chromosomal prophages are significantly enriched in the sequenced virome after growth under unstressed conditions of LF82 (Lennox). Taking into account the copy number of Cyrano episome per LF82 bacteria in this condition, we could not conclude that Cyrano was spontaneously induced on the basis of the virome sequencing. However, it is the set of experiments aimed at this characterization, that allows us to propose that all LF82 prophages are spontaneously induced in Lennox, including Cyrano. These results have been summarized in S2 Tab of the revised manuscript.

4. The authors use a previous Tn-seq analysis to estimate the copy number of Cyrano. Some empirical work must be done to validate these conclusions, as other explanations besides copy number could impact the frequency of Tn insertions in this DNA.

We agree with the reviewer and estimated the Cyrano copy number by relative qPCR, as described in the revised manuscript. We thus estimated, that *E. coli* LF82 carries about 5.5 copies of Cyrano episome per bacterium during the stationary phase, instead of the 3 copies per bacterium estimated using the Tn-Seq analysis. We reviewed the qPCR and virome sequencing analyses based on this result in the revised manuscript.

5. Figure 3: The authors report a relatively high amount of contaminating chromosomal locus DNA from the ybtE gene on p. 9 of the manuscript, however this control is not included in Figure 3, where the authors investigate the impact of antibiotics on phage DNA release into the supernatant. This control needs to be included to validate the conclusions/results.

In order to clarify this point, we put all the data obtained for qPCR analyses (including the bacterial DNA contamination amount) in the new S1 Tab. All figures showing qPCR data were made as follows: if the amount of phages quantified by qPCR is higher than the bacterial DNA contamination, the point is shown in black, otherwise, it is shown in white.

6. How was the indicator strain MAC1403 identified? No mention of this is made in the results section but this is important for evaluating the veracity of the phage isolated from this strain and visualized by TEM. How do the authors know which phages these are? Could they be phages liberated from the indicator strain?

The MAC1403 indicator strain has been identified in a previous study made in our laboratory: “Strain MAC1403 (MG1655 *hsdR*::kanR) is a K12 derivative (phylogroup A) devoid of active prophage and with no DNA restriction activity. It has a lipopolysaccharide (LPS) with a K12-type outer-core component, and is devoid of O-glycosylation (rough phenotype).” DOI: 10.1038/s41467-019-14042-z. As the MAC1403 has been proven to be highly sensitive to phages in our previous study, we used it as a priority to isolate *E. coli* LF82 phages. As described in the materials and methods section, PCR analysis using diagnostic primers identified the phage from the purified plate used for its isolation.

7. The description of how the Galper 1 and 2 phages should be expanded in the results section. How were these hybrid phages isolated? Could they be plaque purified and propagated? How often does hydridization between Gally and Percival happen?

Galper 1 and 2 were isolated on plate thanks to MAC1403 and MAC2225 sensitivity. They are able to be amplified on plate and in liquid culture on MAC1403 and show good titers in this condition (~10^11^ phages/mL after 3h30 of incubation in liquid culture with MAC1403). We observed only two events of Gally-Perceval recombination leading to Galper 1 and 2 that were isolated on plate. Our analysis of the sequencing made on *E. coli* LF82 virome shows that these events are rare (below 4x10^-3^), but lead to the formation of active phages that can be selected and propagated on plate. This information is now included in the revised manuscript in the results and in the material and methods sections.

8. It seems from the way some of the results are described that the authors presume the identify of the phages seen in the supernatants based on predictions related to their genomes, and not actually on any empirical identification criteria. A more detailed description of the work flow used to validate the identity of the phages, which underlies the conclusion that all of the phages are liberated under unstressed conditions and is a main point of the paper, must be included when the authors are describing their results and making conclusions.

Every phages isolated on plate have been identified by PCR amplification thanks to the primers described into Table 3. Perceval, Tritos and Galper 1 and 2 were isolated and identified using this methodology (see pages 27-28).

Gally and Cyrano were not isolated on plate, but present some morphological specificities that allowed us to identify them in the *E. coli* LF82 virome: based on the prophage genomes analysis, Gally is the only podovirus produced by *E. coli* LF82 and Cyrano is the only Siphovirus of a such impressive size. We developed our arguments on pages 9-10.

For clarity, all results concerning the activity of LF82 prophages are resumed in the S2 tab.

9. Figure 5. The authors present hypotheses about the mechanisms of Gally phage replication and propagation and how it may impact the Perceval phage based on sequencing read coverages and changes in the detection of Perceval phage in a strain deleted for Gally phage. The data supports their hypothesis/model, but more experimental work is required to definitively prove the mechanism of replication proposed. The impact of deleting Gally phage on Perceval yield should be compared side-by-side between the wild-type and Gally deletion mutant in the same experiment.

The profile of sequencing read coverage obtained for the Gally prophage and the bacterial chromosome in its vicinity is nearly identical to that observed for its P22 counterpart, for which it is a signature of the P22 phage's replication-packing-excision (RPE) program and its ability to perform lateral transduction (DOI: 10.1038/s41467-021-26520-4). We believe that this observation is sufficient to reasonably propose that Gally uses the same type of program and performs lateral transduction in a manner similar to P22 as well as other *pac* phages endowed with this mechanism. The molecular mechanism of this type of RPE cycle, thought to be dependent on delayed expression of integrase and excisionase (DOI: 10.1126/science.aat5867, DOI: 10.1038/s41467-021-26520-4) relative to the replication machinery remains to be validated for Gally, but we believe this is the subject of another study.

As requested, we show the side-by-side comparison of the number of Perceval DNA-containing particles released from wild-type and ΔGally mutant strains (Figure 5 panel B).

10. The speculation about the mechanism of induction of the phages by antibiotics requires more experimental validation. Why do the authors not investigate the classic induction drug mitomycin C in recA positive and negative backgrounds to confirm their ideas?

Below is presented the result of the Gally production after MMC treatment, when we searched for inducers of Gally at the beginning of the project. MMC (2.5 µg/mL), as ciprofloxacin, increased the production of Gally virions, as expected. In the manuscript, we used ciprofloxacin because it is an antibacterial drug widely used in human clinical applications and in particular to treat patients with active Crohn's disease. This is more relevant than the use of mitomycin C when working with LF82, a microbiota-enriched strain from Crohn's disease patients. Ciprofloxacin is a well-known SOS inducer, like mitomycin C, because it inhibits bacterial topoisomerases and thus induces DNA breaks, which are at the origin of the SOS response (doi: 10.1021/bi5000564). Ciprofloxacin, like mitomycin C, has previously been used for phage induction (e.g. doi: 10.1128/AAC.50 .1.171-177.2006) and we believe that the observed increase in Gally and Cyrano production after ciprofloxacin treatment is sufficient to reasonably propose that Gally and Cyrano are SOS-inducible phages, at least under *in vitro* growth conditions since production of these phages is not increased within macrophages.

Legend: Gally DNA packaged / mL of supernatant after treatment or not with mitomycin C (2.5 µg/mL) (qPCR analysis)

11. Bottom p 15, the authors state that their conclusions about Gally prophage liberation in vitro vs. in macrophages rely on the assumption that the macrophage condition does not affect phage recovery. This should be empirically tested.

We are grateful to the reviewer for raising this important point. Indeed, we had hypothesized in the submitted manuscript that the recovery efficiency of phages was similar from cells grown *in vitro* or within macrophages. We have now tested this assumption by performing a control experiment in which we added a known amount of Gally phage before macrophage lysis, or after filtration of the lysate. We observed an approximately 20-fold decrease in the expected amount of Gally virions after phage recovery and quantification by qPCR 12 hours later. This important result prompted us to re-evaluate the number of Gally virions in macrophage lysates, taking care to respect the same timing as for the control experiment. This factor of loss was taken into account to quantify the Gally virions produced inside the macrophages. The new result and the quantification of Gally loss after their recovery and analysis by qPCR are now included in the revised manuscript and show that the amount of Gally virions produced per bacterium is similar in the unstressed *in vitro* condition and in macrophages.

12. Fig. 7. The authors employ transcriptome analysis across the Gally genome in vitro vs. in macrophages to make conclusions about the viral lifestyle. What is the error in these experiments? Which changes in this figure are significant? Statistical significance is important given there are really no other empirical tests to validate this data.

In response to reviewer 2's comment (see response to point 2, part I), we have modified Figure 7. The gene expression data are now available in S3 and S4 Tab, along with their statistical significance.

13. The authors use a fusion between a viral capsid protein and GFP to visualize induction of lytic growth, however the results are not convincing. The GFP signal is quite diffuse. Further, no work is presented to validate that GFP levels/expressing cells correlate with viral production.

It is well known for the model phages studied so far that major capsid proteins are produced from phage genes that are expressed during the late phase of the lytic cycle. We then thought that the appearance of a fluorescent signal in a strain lysogenized with a Gally prophage encoding a MCP-GFP fusion was sufficient to conclude about the induction of the Gally lytic cycle. The >100-fold increase in the frequency of MCP-GFP-expressing cells after ciprofloxacin treatment compared to unstressed conditions (Fig 8B), and the approximately 20-fold increase in Gally virion production in the wild-type strain after similar treatment (Fig 3), strongly favored our hypothesis regarding the use of the MCP-GFP fusion as a marker for Gally induction. Nevertheless, we added a single-cell time-tracking experiment after ciprofloxacin treatment in the revised version (S4 Fig). Quantification of MCP-GFP-expressing cells and their fate in terms of integrity (lysis or not) leads to the conclusion that nearly all cells that expressed MCP-GFP are lysed before 2.5 h after treatment, in contrast to what is observed for cells that do not express MCP-GFP. This result shows that cells expressing MCP-GFP correlate with cell lysis and thus with the induction of the Gally lytic cycle.

**Part III – Minor Issues: Editorial and Data Presentation Modifications**

Please use this section for editorial suggestions as well as relatively minor modifications of existing data that would enhance clarity.

**Reviewer #1:**

1. It would be beneficial to show whether the Gally deletion enhances the bacterial growth rate in vitro. If it does not significantly affect the overall growth rate of the bacteria, it probably will not affect the survival of E. coli in macrophages regardless of phage induction rate.

As already requested by the reviewer (Part II, reviewer 1, item 1), we added an experiment showing that the presence of Gally greatly affects the survival of LF82 after ciprofloxacin treatment (New Figure 6, panel A and the corresponding paragraph in the Results and Materials and Methods section).

We did not observe any effect of the deletion on the growth rate of bacteria under our *in vitro* growth conditions. This did not allow us to conclude in advance that there was a similar effect in macrophages (putative impact of a modification of the expression of the *torCAD* expression or presence of morons in this condition). For this reason, we tested the survival of the ΔGally mutant in macrophages. The other reason was to verify the absence of a massive induction of Gally in macrophages that could affect the survival of the bacteria.

2. Pages 2, 5, and 11: “.. show that all of them form virions”, “We show that the five prophages form virions in vitro.”: The authors failed to detect Cartapus virions. So then I wonder how they concluded that “the five LF82 prophages are induced and form virions.”

Cartapus virions are detectable in the virome produced by LF82 after growth in Lennox, as shown by qPCR analysis (Fig 3) and DNA sequencing (Fig 2). We have added a statistical analysis in the revised manuscript showing that all integrated prophages are significantly enriched in the sequenced virome of unstressed LF82 bacteria grown in Lennox, including Cartapus. Finally, it is the set of experiments aimed at their characterization that allows us to propose that all LF82 prophages are spontaneously induced in Lennox. These results have been summarized in S2 Tab of the revised manuscript.

3. Page 11: “Gally could not be propagated under all conditions tested.”: Is it possible that Gally does not lyse cells upon induction?

This is a very good question that we spent a lot of time on. It is clear that when the Gally prophage is present in the chromosome, its induction with ciprofloxacin leads to lysis of its host, as indicated by the OD_600_ drop in culture (see new panel A in Figure 6) of the wild-type strain but not (at least not at the same level) of the ΔGally strain. The simplest explanation is that Gally endolysins are responsible for this observation. However, when Gally virions are used to infect different strains, including the ΔGally mutant, no plaque was observed. Using a Gally prophage containing a kanR cassette, we now show that Gally particles are able to infect the and lysogenize a ΔGally strain, giving colonies on LB agar supplemented with kanamycin. This important result, included in the revised manuscript, demonstrates that Gally virions are capable of infecting the ΔGally strain. The absence of plaque detection is either due to a very small plaque size, or to a lysis / lysogeny choice heavily tilted towards lysogenization under our test conditions. We do not know why at present, but the mechanism that regulates the Gally lysis/lysogeny balance will be the subject of future study.

4. Page 5: explain what PhoP is.

Explanation is given in the introduction of the revised manuscript.

5. Pages 7,9,25: NMDM -> DMEM?

Correction done.

6. Table 1 and page 9: Bor transcription was increased 50-fold above background in LB but not listed in Table 1. However, the table title says it lists prophages genes showing 5-fold or higher expression in LB OR DMEM, not AND.

We thank the reviewer's question on *bor* that allowed us to detect a mistake in the submitted manuscript. *bor* was overexpressed 50 times above the background in DMEM media but not in LB, which explains its absence in Table 1 (in fact, we wrote « or » but it was « and »). From these observations, it is clear that morons must be defined under particular growth conditions and may vary under different conditions, as is the case for *bor*. To avoid misinterpretation of our analyses, we decided to show only the morons expressed in LB medium. These genes are shown in the revised Table 1 and have been identified using reprocessed RNAseq data displayed in the new S4 Tab.

7. Fig. 2 and 5: the light gray parts are hard to see.

To avoid confusion between the coverage of the reads corresponding to the prophage and those corresponding to the bacterial chromosome we have deliberately accentuated the difference between the grey parts.

**Reviewer #2:**

1. P5, L13-L21(related to ref. 15): In this case, lambda is induced in macrophages in a RecA-independent / phoP-dependent mechanism (by membrane stress induced by the mCramp1 anti-microbial peptide, ). It is not clear for me how the authors consider such kinds of stresses as potential factors for prophage induction.

The reviewer is absolutely right. We don’t know whether this *phoP* regulation applies to different prophage species such as Gally, but in any case, *phoP* is expressed in phagocyted LF82 bacteria (S4 tab), at least at the global population level. So we can exclude the hypothesis that the repression of the Gally lytic cycle is due to a defect in PhoP. We have added this observation in the discussion section of the revised manuscript.

2. P7, L3 and P24 (prophage content of LF82): Does this strain contain degraded prophages/prophage remnants? How many prophage-like regions were predicted by PHASTER? In the encapsidated DNA samples, did the authors detect significant numbers of reads from some of these regions?

As indicated in the Material and Methods section, we searched systematically for the prophage content of LF82 using PHASTER (which is rather over-predicting). The output shown below indicates that no other phages, complete or not, was identified. We now added this observation in the result section. In the encapsidated DNA of the LF82 virome, we observed a background of bacterial DNA (which can come from contamination or transduction), over which the 4 prophage regions peak (more or less), as well as the lateralized transduced region downstream of Gally.

3. P7, L15-17: As the authors reannotated the 5 prophages, please provide a supplementary table to show the RNA seq data for the 5 prophage regions, including the "local background" of each prophage region and the data in macrophages. I looked at the data presented in Supplementary Data 2 in Ref. 26, but it is not easy to understand how the current results are related to the data presented in the Supplementary Data 2.

The corresponding S4 Tab (for all genes of LF82) and S3 Tab (information only for prophages) have been added in the revised manuscript. It contains all transcriptomic data from Prudent et al (DOI: 10.1038/s42003-021-02161-7), that have been re-processed as indicated in the material and method section. It contains the newly annotation of all phage genes and a comparison of normalized gene expression between the LB and macrophages growth conditions.

4. P10, L14-L15 (concentration of Gally genome): Considering the chromosome DNA contamination level, the actual encasidated Gally genome seems to be much lower.

As indicated in the manuscript, we estimated by qPCR an average bacterial contamination value for the virome preparation of 2.3x10^4^ chromosomal copies/mL. Since the Gally prophage is present in only one copy per bacterial chromosome, this contamination is negligible compared to the number of Gally genome measured in the virome preparation (4.3x10^8^ Gally genomes/mL). This implies that the 4.3x10^8^ Gally genomes/mL represent almost exclusively encapsidated Gally genomes.

5. P14, L2-3 (antibiotics treatment, 2 hr induction): Why did the authors not use MMC? Was MMC treatment less effective in the induction of LF82 prophages? Why and how did the authors decide to employ this condition (2 hr treatment)? Clear cell lysis or some other signs of phage induction were observed at this time point? I feel that 2 hr is too short to achieve the maximum induction level.

For the use of ciprofloxacin instead of MMC, see the answer to reviewer 3's point 10 in Part II. We have added an experiment showing ciprofloxacin-dependent lysis of wild-type LF82 bacteria over time (Figure 6 panel A). This experiment shows that bacterial lysis under ciprofloxacin treatment is primarily due to Gally and begins about 50 min after the addition of ciprofloxacin. Cell lysis appears to be complete after about 90 min. We believe that a 2 hours treatment is then sufficient to obtain the maximum phage production.

6. P15, 10 (leftward region) and Figure 7: The region from c2 to the last gene before xis is not a replication module. Related to this, although the gene map of Gally is presented in Figure 1, please show it (the gene organization as "prophage") in this figure to make it easier for readers to understand the data in Figure 7 in the context of gene organization.

Correction done. We have modified the Figure 7 in the revised manuscript and shown the gene organization of Gally as requested.

7. P15, L13-L18 (mRNA level of regulators): In both samples, phage induction occurred only in a very small fraction of bacterial cells. It seems that the authors do not consider this point, at least here.

The reviewer is right, if we consider the Gally genes involved in the lytic cycle. Indeed, we know that about 0.04% of bacteria induce the expression of MCP and probably all genes involved in the Gally lytic cycle. The overexpression of these genes is barely visible in the transcriptomic analysis (Fig 7, S3 Tab). In contrast, we do not know what fraction of the bacteria could induce Gally regulatory genes, such as Mnt, in macrophages. If a specific regulatory mechanism in macrophages involved Gally repressors, it could be that these genes are induced in a significant fraction of bacteria within macrophages.

8. P15, the second last line (2 to 5x106): I can not understand how this value was obtained.

Since we have reevaluated the number of Gally virions produced in macrophages (see response to reviewer 3, point 11 part II), this paragraph has changed in the revised version.

9. P25, L19: Does the SOS response in macrophages peaks at 6-hr P.I.? In my understanding (according to Ref. 24), SOS response starts at around 6-hr P.I.

The reviewer has right. We modified the sentence.

Additional more minor comments:

P4, L5 & L10: Refs 2-5 are adequate and required for "up to 70% of strains in species of the human microbiota"? Ref. 10 is related to "metabolic genes"? Refs 11 & 12 (identification of lom and bor) are adequate for "adaptation to a given environment"?

Indeed, references 2-5 identify lysogens present in mouse and human intestinal bacteria, not only strains of the human microbiota. We modify the sentence accordingly. In Ref 10, the presence of *sodC* in the phage gifsy 2 is an example of a phage metabolic gene. Lom and Bor (Ref 11 and 12) are respectively involved in the ability of *E. coli* strains to interact with and invade human epithelial cells and in the resistance of lysogenized *E. coli* to killing by serum.

P7, L5 (prophage names): Why and how did the authors give specific names to the five prophages? This is rather a personal question, but interesting for me.

The authors followed the new recommendations of the ICTV subcomitee for archeal and bacterial viruses. These recommandations require to name phages with specific names, by avoiding using simply number or P+number. Following this guideline, names were given to the phages by the students that worked on them. Gally is from the Manga "Gunnm", Perceval is the famous knight from King Arthur's tales, Cyrano from Edmond Rostand's play (that choice was motivated by Cyrano's size genome, comparable to Cyrano's nose size in the play). The name Tritos comes from Greek and means 3. Cartapus is a spy character in the French movie, “Astérix et Obélix : Mission Cléopâtre”. It refers to a famous line, when Cartapus the spy explains to Caesar her technique to infiltrated the enemy in the greatest secret. As we detected Cartapus after virome sequencing, but not in every replicates in the qPCR analysis, we deduced its relationship to this famous spy. Galper is a fusion between the names Gally and Perceval. Indeed, Galper is the result of recombination between these two prophages. Science is fun…

P8, Table 1 title (or): Should be "and".

The Table 1 in the revised version has changed and highlights moron identified only in LB medium (overnight culture). See response to reviewer 1, point 6 part III.

P9, the last line (180kb): Please explain what this is.

This number referred to the distance between the end of the Gally prophage and the start of the Perceval prophage. After reviewing the prophage boundaries this number was changed to 135 kb instead of 180.

P7, L10-11,14 (HK620, P22, Fels2): Please cite appropriate references for the readers who are not so familiar with these phages.

We added references for these three phages.

P11, L2 & P17, L18-L20 (failure in the isolation of Gally and infectivity of Gally): Can Gally adsorb to LF82 cells? As the Gally-deleted LF82 is not sensitive to Gally, Gally may be no more infective (defective in terms of infectivity) due to some mutation(s) in tail genes (or Gally might be acquired by LF82 by some mechanism other than normal infection).

See response to reviewer 1, point 3, part III. We show now that Gally can infect the ΔGally strain but is unable to form plaques in our test condition. This is indicated in the revised manuscript.

P18, L1 (highly): Should be " widely" or " frequently"

Correction done.

P19, L6 (hyperactive): Gally is active, but it can not be said that it is hyperactive.

Correction done, we changed to “highly produced”.

**Reviewer #3:** (No Response)
